# A bibliometric and Altmetric analysis of the 100 top most cited articles on dentin adhesives

**DOI:** 10.1007/s00784-024-05498-5

**Published:** 2024-01-13

**Authors:** Ferda Karabay, Mustafa Demirci, Safa Tuncer, Neslihan Tekçe, Meriç Berkman

**Affiliations:** 1 Deparment of Restorative Dentistry, Faculty of Dentistry, Istanbul Medeniyet University, Istanbul, Turkey; 2 Deparment of Restorative Dentistry, Faculty of Dentistry, Istanbul University, Istanbul, Turkey; 3 Deparment of Restorative Dentistry, Faculty of Dentistry, Kocaeli University, Kocaeli, Turkey; 4 Deparment of Restorative Dentistry, Faculty of Dentistry, Yeditepe University, Istanbul, Turkey

**Keywords:** Bibliometric, Altmetric, Citation analysis, Dentin adhesive, Restorative dentistry

## Abstract

**Objective:**

This study aimed to identify the 100 top-cited articles on dentin adhesives utilizing comprehensive bibliometric and altmetric analyses.

**Materials and methods:**

The Institute of Scientific Information Web of Knowledge database was used to compile the top-cited articles published from 1945 through February 12, 2023. Citation counts were manually retrieved for each article from Scopus, Google Scholar, Dimensions, and Altmetric. The articles were analyzed in terms of their number of citations, year, journal name, author (name, institution, and country), and type and specific field of study. We used descriptive statistics to summarize the results.

**Results:**

The analysis revealed that the top 100 cited articles originated from 18 English-language journals and collectively accumulated a remarkable 34526 citations. The article with the highest number of citations garnered 1288 references. Among authors, Van Meerbeek B. stood out with nine articles and 4650 citations, followed by Pashley D.H. with six articles and 2769 citations. Japan was the leading contributor by country, while the Catholic University of Leuven led in terms of institutions with 20 articles.

**Conclusion:**

According to this study, basic research and review articles garnered the most citations, respectively. The citation analysis revealed different trends for researchers, the first being that researchers have focused on basic fields such as the ultramorphology of dentin and adhesive interfaces, followed by bond strength to dentin. Two studies on clinical experiences suggested that studies with high-level evidence, such as systematic reviews, meta-analyses, or randomized controlled clinical trials, are required.

**Clinical relevance:**

It is identified that more studies with high-level evidence-based research are needed in the field of dental adhesives.

**Supplementary Information:**

The online version contains supplementary material available at 10.1007/s00784-024-05498-5.

## Introduction

Bibliometrics is one of the few subfields involved in the measurement of science outputs [1]. Bibliometric indicators are useful tools for evaluating research performance, provided they are precise, advanced, up-to-date, combined with expert knowledge, and interpreted and applied with care [2]. Citation analysis is a principal bibliometric approach [2]. Citations may not fully reflect the quality of a work, but highly cited articles often present new ideas or address important problems, so they are valuable in the scientific world. Additionally, the frequent citation of an article could be a strong indication of its reliability as a source for researchers to substantiate their methods or arguments [3].

Since 1945, the Institute of Scientific Information (ISI) has been collecting bibliometric data from published scientific papers, but their collection was not launched until the Science Citation Index (SCI), a special tool for measuring citations, was first published in 1962 [4]. Today, the most widely used databases for bibliometric studies are the citation indexes produced by Thomson Reuters, especially Web of Science (WoS) and its predecessor, the SCI [2]. Google Scholar, a tool sponsored by the Internet search company Google, was created to provide users with a simple way of searching a broad range of scientific literature. Google Scholar employs a matching algorithm to search for keyword search terms in the title, summary, or full text of an article from various publishers and websites [5]. Around the same time Google Scholar was announced to the public, Elsevier introduced Scopus, an indexing and abstraction service that includes its own citation-tracking tool. Scopus has reportedly indexed more journals than WoS has and included more international and open-access journals [5].

Altmetric (https://www.altmetric.com) is powered by Digital Science, a Macmillan company that focuses on technology to aid scientific research. It collects data from three primary sources: social media (e.g., Twitter, Facebook, Google, Pinterest, and blogs); traditional media, both mainstream (e.g., *The Guardian* and *New York Times*) and science-specific (e.g., *New Scientist* and *Scientific American*); and online reference managers (e.g., Mendeley and CiteULike). It also calculates the score of an article on the basis of its wager on those sources. This is an algorithm-calculated quantitative measure of the article's quality and amount of attention [6].

In early 2018, Digital Science & Research Solutions launched Dimensions, a novel online academic platform designed to provide a distinct viewpoint on research outcomes. Grant awards, journal and book publications, mentions of social media, academic citations, clinical trials, and commercial patents are considered research outputs. The publication and citation contents at Dimensions are created and constantly updated by integrating data from multiple sources, including multiple clinical trial records, open-access articles, indexes covering many scientific journals, databases with content licenses, and open-access databases [7].

Numerous citation analyses and the most cited articles have become available in dentistry, including areas such as caries [8, 9], bulk-fill composites [10], endodontics [11–17], implants [18–20], pediatric dentistry [21, 22], periodontology [23, 24], oral medicine and radiology [25–27], and orthodontics [28], and in topics such as dental traumatology [12, 29], tooth wear [30], minimally invasive dentistry [31], orofacial pain [32], and dental education [33]. Some citation analysis studies have included articles published in multiple dentistry journals [4, 34–38] or in a single dentistry journal [13, 39].

Dentin adhesives appear to have made tremendous progress over the years since adhesives were first introduced in 1955 by Buonocore in a study on the bonding of resins to etched enamel surfaces and later after the introduction of resin bondings to adhere to etched dentin by Fusuyama et al. [40–42]. Dental adhesive technology is constantly evolving with the rapid changes in commercial adhesives. These developments are the result of numerous laboratory and clinical studies, and the data obtained are highly important in showing the potential success of these materials and in guiding future research [43].

The basic mechanism of bonding to enamel and dentin involves the replacement of resin monomers with the minerals removed from the dental hard tissues, which cause porosity, and upon setting, micromechanical interlocking occurs in the formed porosities [44]. Adhesives can be classified as “etch and rinse” or “self-etching” depending on the underlying adhesion strategy, and the degree of substance exchange varies significantly among these adhesives [44]. Nevertheless, the success of both adhesion strategies has been reported in both laboratory and clinical research. However, it’s important to note that their effectiveness may depend on the specific product being used [45].

To date, no bibliometric analysis has been carried out to provide a more comprehensive perspective to evaluate research on various topics in the field of dentin adhesives, enabling us to anticipate future advancements and direct research efforts in this area. Thus, the purposes of this study were to gain insight into the scientific interests, research trends, and development within the field of dental adhesives by using WoS, Scopus, Google Scholar, Altmetric, and Dimensions.

## Materials and methods

To identify the most cited articles on dentin adhesives, our study was conducted in two stages, in which bibliometric and altmetric analysis data were collected. Institutional ethics committee approval was not necessary because the data used in this study were obtained from publications.

Initially, the WoS database (http://www.webofknowledge.com) was used for the bibliometric analysis. On February 12, 2023, a search was conducted in the "Web of Science Core Collection (WoSCC)" using the search terms listed in Table S1, starting from the year 1945. The most commonly used free and Medical Subject Headings (MeSH) terms in the published literature on dentin adhesives were combined to create keywords. The field tags as “Topic” were selected, and the search resulted in 142,494 articles ranked according to the first option with the highest number of citations. Then, respectively, the search was restricted to articles written in the English language (*n* = 137,996), and ‘Science Citation Index Expanded (SCI-E)’ and ‘Emerging Science Citation Index (ESCI)’ index limitations were applied, resulting in 123,086 articles. The document types “article” and “review article” were selected (*n* = 115,845). After screening the articles, all studies were exported into the Excel program as a full record.

After ranking the articles according to their numbers of citations in the WoS database, two independent researchers (F.K. and M.D.) reviewed the titles and abstracts of the articles to identify the candidates for full-text review. Apart from the restrictions set, the eligibility criteria consisted mainly of studies that had data or topics that directly included dentin adhesives. The first 100 articles with the highest number of citations according to the criteria were identified independently by the two researchers (F.K. and M.D.). All results were cross-checked, and inconsistencies were resolved after reading the full texts of the articles and reviewing the relevant literature. The inter-examiner agreement was quantified using the kappa coefficient.

After the top 100 most cited articles were identified, the citation counts were manually retrieved for each article from the Scopus (https://www.scopus.com/), Google Scholar (https://scholar.google.com), and Dimensions databases (https://app.dimensions.ai) on the same date to provide a more comprehensive view, as the citation count of the same article may vary on different dates (date of access: March 3, 2023).

For the altmetric analysis, the Altmetric Attention Score (AAS; a metric that automatically calculates the weighted count of social media attention received by a research output) was used. The 100 most cited articles were accessed by manually scanning the Altmetric Explorer database (https://www.altmetric.com) through the “Advanced Search” option using “publication title” or “DOI” simultaneously (date of access: March 3, 2023). A donut graph with different colors representing the amount of attention given to the different types of output was constructed with the AASs. Articles that were found in the database but were not cited in other articles and those that were added to the database either by institutional implementation or through a non-scoring source were displayed in the donut with a question mark. If the article was not mentioned at all in any article or if this output did have a score at one point but had been removed/reduced because of changes in the number of mentions, it was represented with “0” in the altmetric donut. At this point, there would be no difference in that both cases would indicate having no tracked attention or altmetric score assigned to the research output (help.altmetric.com).

The top 100 most cited articles are shown in Table 1 according to their numbers of citations as indicated in the WoSCC database, from highest to lowest, including results from all databases searched. As the numbers of citations were the same, our top 100 list consisted of 101 articles. After the final list was confirmed, the top 100 most cited articles were analyzed by the researchers, who recorded the number of citations, publication name (title), year of publication, journal name and impact factor, author(s) (name, number, and authorship position), country, institution, and type and field of study. When the article analysis results were discrepant between the two independent researchers, a consensus decision was reached through a discussion.

**Table 1:** The 100 most cited articles on dentin adhesives

Rank	Article	No. of citatitons					
		WOS		Scopus	Google scholar	Dimension	AAS
		Total	Avg. per year				
1	Van Meerbeek B, De Munck J, Yoshida Y, Inoue S, Vargas M, Vijay P, Van Landuyt K, Lambrechts P, Vanherle G. Buonocore memorial lecture. Adhesion to enamel and dentin: current status and future challenges. Oper Dent. 2003 May-Jun;28(3):215–35	1288	64.4	1464	3118	1100	0
2	De Munck J, Van Landuyt K, Peumans M, Poitevin A, Lambrechts P, Braem M, Van Meerbeek B. A critical review of the durability of adhesion to tooth tissue: methods and results. J Dent Res. 2005 Feb;84(2):118–32	1200	66.67	1345	2836	1214	3
3	Van Landuyt KL, Snauwaert J, De Munck J, Peumans M, Yoshida Y, Poitevin A, Coutinho E, Suzuki K, Lambrechts P, Van Meerbeek B. Systematic review of the chemical composition of contemporary dental adhesives. Biomaterials. 2007 Sep;28(26):3757–85	887	55.44	963	1759	911	9
4	Breschi L, Mazzoni A, Ruggeri A, Cadenaro M, Di Lenarda R, De Stefano Dorigo E. Dental adhesion review: aging and stability of the bonded interface. Dent Mater. 2008 Jan;24(1):90–101	822	54.8	885	1707	850	24
5	Van Meerbeek B, Yoshihara K, Yoshida Y, Mine A, De Munck J, Van Landuyt KL. State of the art of self-etch adhesives. Dent Mater. 2011 Jan;27(1):17–28	817	68..08	899	1702	863	8
6	Yoshida Y, Nagakane K, Fukuda R, Nakayama Y, Okazaki M, Shintani H, Inoue S, Tagawa Y, Suzuki K, De Munck J, Van Meerbeek B. Comparative study on adhesive performance of functional monomers. J Dent Res. 2004 Jun;83(6):454–8	777	40.89	851	1477	833	0
7	Pashley DH, Tay FR, Yiu C, Hashimoto M, Breschi L, Carvalho RM, Ito S. Collagen degradation by host-derived enzymes during aging. J Dent Res. 2004 Mar;83(3):216–21	709	37.32	785	1363	763	7
8	Pashley DH, Tay FR, Breschi L, Tjäderhane L, Carvalho RM, Carrilho M, Tezvergil-Mutluay A. State of the art etch-and-rinse adhesives. Dent Mater. 2011 Jan;27(1):1–16	620	51.67	679	1271	704	4
9	Sano H, Shono T, Sonoda H, Takatsu T, Ciucchi B, Carvalho R, Pashley DH. Relationship between surface area for adhesion and tensile bond strength–evaluation of a micro-tensile bond test. Dent Mater. 1994 Jul;10(4):236–40	603	20.79	675	1386	601	3
10	Peumans M, Kanumilli P, De Munck J, Van Landuyt K, Lambrechts P, Van Meerbeek B. Clinical effectiveness of contemporary adhesives: a systematic review of current clinical trials. Dent Mater. 2005 Sep;21(9):864–81	582	32.33	647	1225	606	5
11	Tay FR, Pashley DH. Aggressiveness of contemporary self-etching systems. I: Depth of penetration beyond dentin smear layers. Dent Mater. 2001 Jul;17(4):296–308	509	23.14	581	1094	504	0
12	Sano H, Takatsu T, Ciucchi B, Horner JA, Matthews WG, Pashley DH. Nanoleakage: leakage within the hybrid layer. Oper Dent. 1995 Jan-Feb;20(1):18–25	501	17.89	550	988	418	0
13	Van Meerbeek B, Peumans M, Poitevin A, Mine A, Van Ende A, Neves A, De Munck J. Relationship between bond-strength tests and clinical outcomes. Dent Mater. 2010 Feb;26(2):e100-21	499	38.38	531	1110	496	5
14	Van Meerbeek B, Inokoshi S, Braem M, Lambrechts P, Vanherle G. Morphological aspects of the resin-dentin interdiffusion zone with different dentin adhesive systems. J Dent Res. 1992 Aug;71(8):1530–40	488	15.74	510	887	373	6
15	Hashimoto M, Ohno H, Kaga M, Endo K, Sano H, Oguchi H. In vivo degradation of resin-dentin bonds in humans over 1 to 3 years. J Dent Res. 2000 Jun;79(6):1385–91	477	20.74	531	934	471	3
16	Tay FR, Pashley DH, Yoshiyama M. Two modes of nanoleakage expression in single-step adhesives. J Dent Res. 2002 Jul;81(7):472–6	475	22.62	512	766	449	3
17	De Munck J, Van Meerbeek B, Yoshida Y, Inoue S, Vargas M, Suzuki K, Lambrechts P, Vanherle G. Four-year water degradation of total-etch adhesives bonded to dentin. J Dent Res. 2003 Feb;82(2):136–40	463	23.15	504	912	440	0
18	Tay FR, Pashley DH, Suh BI, Carvalho RM, Itthagarun A. Single-step adhesives are permeable membranes. J Dent. 2002 Sep-Nov;30(7–8):371–82	462	22	496	924	446	0
19	Moszner N, Salz U, Zimmermann J. Chemical aspects of self-etching enamel-dentin adhesives: a systematic review. Dent Mater. 2005 Oct;21(10):895–910	457	25.39	502	898	491	9
20	Ito S, Hashimoto M, Wadgaonkar B, Svizero N, Carvalho RM, Yiu C, Rueggeberg FA, Foulger S, Saito T, Nishitani Y, Yoshiyama M, Tay FR, Pashley DH. Effects of resin hydrophilicity on water sorption and changes in modulus of elasticity. Biomaterials. 2005 Nov;26(33):6449–59	431	23.94	468	737	459	6
21	Pashley DH, Carvalho RM. Dentine permeability and dentine adhesion. J Dent. 1997 Sep;25(5):355–72	408	15.69	446	1006	406	0
22	Labella R, Lambrechts P, Van Meerbeek B, Vanherle G. Polymerization shrinkage and elasticity of flowable composites and filled adhesives. Dent Mater. 1999 Mar;15(2):128–37	404	16.83	452	970	411	0
23	Van Meerbeek B, Perdigão J, Lambrechts P, Vanherle G. The clinical performance of adhesives. J Dent. 1998 Jan;26(1):1–20	400	16	444	1037	338	3
24	Pashley DH, Sano H, Ciucchi B, Yoshiyama M, Carvalho RM. Adhesion testing of dentin bonding agents: a review. Dent Mater. 1995 Mar;11(2):117–25	393	14.04	428	970	376	?
25	Pashley DH, Tay FR. Aggressiveness of contemporary self-etching adhesives. Part II: etching effects on unground enamel. Dent Mater. 2001 Sep;17(5):430–44	383	17.41	444	862	373	0
26	Hebling J, Pashley DH, Tjäderhane L, Tay FR. Chlorhexidine arrests subclinical degradation of dentin hybrid layers in vivo. J Dent Res. 2005 Aug;84(8):741–6	381	21.17	427	782	426	0
27	Carrilho MR, Geraldeli S, Tay F, de Goes MF, Carvalho RM, Tjäderhane L, Reis AF, Hebling J, Mazzoni A, Breschi L, Pashley D. In vivo preservation of the hybrid layer by chlorhexidine. J Dent Res. 2007 Jun;86(6):529–33	380	23.75	423	733	425	0
28	Sano H, Yoshikawa T, Pereira PN, Kanemura N, Morigami M, Tagami J, Pashley DH. Long-term durability of dentin bonds made with a self-etching primer, in vivo. J Dent Res. 1999 Apr;78(4):906–11	370	15.42	417	708	363	0
29	Malacarne J, Carvalho RM, de Goes MF, Svizero N, Pashley DH, Tay FR, Yiu CK, Carrilho MR. Water sorption/solubility of dental adhesive resins. Dent Mater. 2006 Oct;22(10):973–80	363	21.35	409	728	402	3
30	Goracci C, Tavares AU, Fabianelli A, Monticelli F, Raffaelli O, Cardoso PC, Tay F, Ferrari M. The adhesion between fiber posts and root canal walls: comparison between microtensile and push-out bond strength measurements. Eur J Oral Sci. 2004 Aug;112(4):353–61	358	18.84	418	856	432	0
31	Bouillaguet S, Troesch S, Wataha JC, Krejci I, Meyer JM, Pashley DH. Microtensile bond strength between adhesive cements and root canal dentin. Dent Mater. 2003 May;19(3):199–205	356	17.8	410	807	380	0
32	Van Meerbeek B, Willems G, Celis JP, Roos JR, Braem M, Lambrechts P, Vanherle G. Assessment by nano-indentation of the hardness and elasticity of the resin-dentin bonding area. J Dent Res. 1993 Oct;72(10):1434–42	356	11.87	388	640	315	?
33	Imazato S. Antibacterial properties of resin composites and dentin bonding systems. Dent Mater. 2003 Sep;19(6):449–57	326	16.3	349	575	342	8
34	Spencer P, Wang Y. Adhesive phase separation at the dentin interface under wet bonding conditions. J Biomed Mater Res. 2002 Dec 5;62(3):447–56	324	15.43	347	500	321	?
35	Watanabe I, Nakabayashi N, Pashley DH. Bonding to ground dentin by a phenyl-P self-etching primer. J Dent Res. 1994 Jun;73(6):1212–20	315	10.86	369	653	296	3
36	Van Landuyt KL, De Munck J, Snauwaert J, Coutinho E, Poitevin A, Yoshida Y, Inoue S, Peumans M, Suzuki K, Lambrechts P, Van Meerbeek B. Monomer-solvent phase separation in one-step self-etch adhesives. J Dent Res. 2005 Feb;84(2):183–8	310	17.22	347	565	310	0
37	Spencer P, Ye Q, Park J, Topp EM, Misra A, Marangos O, Wang Y, Bohaty BS, Singh V, Sene F, Eslick J, Camarda K, Katz JL. Adhesive/Dentin interface: the weak link in the composite restoration. Ann Biomed Eng. 2010 Jun;38(6):1989–2003	308	23.69	325	531	347	6
38	Scherrer SS, Cesar PF, Swain MV. Direct comparison of the bond strength results of the different test methods: a critical literature review. Dent Mater. 2010 Feb;26(2):e78-93	303	23.31	326	657	300	?
39	Tay FR, Pashley DH. Water treeing–a potential mechanism for degradation of dentin adhesives. Am J Dent. 2003 Feb;16(1):6–12	302	15.1	325	595	309	0
40	Nakabayashi N, Takarada K. Effect of HEMA on bonding to dentin. Dent Mater. 1992 Mar;8(2):125–30	302	9.74	318	583	297	3
41	Van Meerbeek B, Dhem A, Goret-Nicaise M, Braem M, Lambrechts P, VanHerle G. Comparative SEM and TEM examination of the ultrastructure of the resin-dentin interdiffusion zone. J Dent Res. 1993 Feb;72(2):495–501	296	9.87	311	516	223	3
42	Tjäderhane L, Nascimento FD, Breschi L, Mazzoni A, Tersariol IL, Geraldeli S, Tezvergil-Mutluay A, Carrilho MR, Carvalho RM, Tay FR, Pashley DH. Optimizing dentin bond durability: control of collagen degradation by matrix metalloproteinases and cysteine cathepsins. Dent Mater. 2013 Jan;29(1):116–35	291	29.1	339	507	335	3
43	Geurtsen W. Biocompatibility of resin-modified filling materials. Crit Rev Oral Biol Med. 2000;11(3):333–55	290	12.61	319	536	321	0
44	Mazzoni A, Pashley DH, Nishitani Y, Breschi L, Mannello F, Tjäderhane L, Toledano M, Pashley EL, Tay FR. Reactivation of inactivated endogenous proteolytic activities in phosphoric acid-etched dentine by etch-and-rinse adhesives. Biomaterials. 2006 Sep;27(25):4470–6	286	16.82	321	500	301	0
45	Hashimoto M, Ohno H, Sano H, Kaga M, Oguchi H. In vitro degradation of resin-dentin bonds analyzed by microtensile bond test, scanning and transmission electron microscopy. Biomaterials. 2003 Sep;24(21):3795–803	285	14.25	302	522	277	0
46	Eick JD, Gwinnett AJ, Pashley DH, Robinson SJ. Current concepts on adhesion to dentin. Crit Rev Oral Biol Med. 1997;8(3):306–35	285	10.96	319	589	261	3
47	Carrilho MR, Carvalho RM, de Goes MF, di Hipólito V, Geraldeli S, Tay FR, Pashley DH, Tjäderhane L. Chlorhexidine preserves dentin bond in vitro. J Dent Res. 2007 Jan;86(1):90–4	279	17.44	316	613	321	3
48	Rosa WL, Piva E, Silva AF. Bond strength of universal adhesives: A systematic review and meta-analysis. J Dent. 2015 Jul;43(7):765–76	276	34.5	324	637	343	12
49	Orchardson R, Gillam DG. Managing dentin hypersensitivity. J Am Dent Assoc. 2006 Jul;137(7):990–8; quiz 1028–9	274	16.12	359	778	336	9
50	Sarrett DC. Clinical challenges and the relevance of materials testing for posterior composite restorations. Dent Mater. 2005 Jan;21(1):9–20	274	15.22	285	582	302	9
51	Wang Y, Spencer P. Hybridization efficiency of the adhesive/dentin interface with wet bonding. J Dent Res. 2003 Feb;82(2):141–5	274	13.7	296	489	273	3
52	Nakajima M, Sano H, Burrow MF, Tagami J, Yoshiyama M, Ebisu S, Ciucchi B, Russell CM, Pashley DH. Tensile bond strength and SEM evaluation of caries-affected dentin using dentin adhesives. J Dent Res. 1995 Oct;74(10):1679–88	274	9.79	299	549	260	0
53	Goldberg M. In vitro and in vivo studies on the toxicity of dental resin components: a review. Clin Oral Investig. 2008 Mar;12(1):1–8	268	17.87	295	503	306	4
54	Nishitani Y, Yoshiyama M, Wadgaonkar B, Breschi L, Mannello F, Mazzoni A, Carvalho RM, Tjäderhane L, Tay FR, Pashley DH. Activation of gelatinolytic/collagenolytic activity in dentin by self-etching adhesives. Eur J Oral Sci. 2006 Apr;114(2):160–6	267	15.71	298	464	274	0
55	Versluis A, Tantbirojn D, Douglas WH. Why do shear bond tests pull out dentin? J Dent Res. 1997 Jun;76(6):1298–307	262	10.08	279	510	240	0
56	Pashley DH, Tay FR, Carvalho RM, Rueggeberg FA, Agee KA, Carrilho M, Donnelly A, García-Godoy F. From dry bonding to water-wet bonding to ethanol-wet bonding. A review of the interactions between dentin matrix and solvated resins using a macromodel of the hybrid layer. Am J Dent. 2007 Feb;20(1):7–20	256	16	280	442	267	0
57	Tjäderhane L, Nascimento FD, Breschi L, Mazzoni A, Tersariol IL, Geraldeli S, Tezvergil-Mutluay A, Carrilho M, Carvalho RM, Tay FR, Pashley DH. Strategies to prevent hydrolytic degradation of the hybrid layer-A review. Dent Mater. 2013 Oct;29(10):999–1011	251	25.1	283	450	283	3
58	Van Meerbeek B, Van Landuyt K, De Munck J, Hashimoto M, Peumans M, Lambrechts P, Yoshida Y, Inoue S, Suzuki K. Technique-sensitivity of contemporary adhesives. Dent Mater J. 2005 Mar;24(1):1–13	251	13.94	270	555	256	0
59	Ratanasathien S, Wataha JC, Hanks CT, Dennison JB. Cytotoxic interactive effects of dentin bonding components on mouse fibroblasts. J Dent Res. 1995 Sep;74(9):1602–6	251	8.96	269	450	252	3
60	Dietschi D, Duc O, Krejci I, Sadan A. Biomechanical considerations for the restoration of endodontically treated teeth: a systematic review of the literature, Part II (Evaluation of fatigue behavior, interfaces, and in vivo studies). Quintessence Int. 2008 Feb;39(2):117–29	246	16.4	257	653	242	0
61	Kanemura N, Sano H, Tagami J. Tensile bond strength to and SEM evaluation of ground and intact enamel surfaces. J Dent. 1999 Sep;27(7):523–30	245	10.21	286	496	242	0
62	Inoue S, Koshiro K, Yoshida Y, De Munck J, Nagakane K, Suzuki K, Sano H, Van Meerbeek B. Hydrolytic stability of self-etch adhesives bonded to dentin. J Dent Res. 2005 Dec;84(12):1160–4	244	13.56	255	422	242	0
63	Sano H, Shono T, Takatsu T, Hosoda H. Microporous dentin zone beneath resin-impregnated layer. Oper Dent. 1994 Mar-Apr;19(2):59–64	244	8.41	259	460	200	0
64	Breschi L, Mazzoni A, Nato F, Carrilho M, Visintini E, Tjäderhane L, Ruggeri A Jr, Tay FR, Dorigo Ede S, Pashley DH. Chlorhexidine stabilizes the adhesive interface: a 2-year in vitro study. Dent Mater. 2010 Apr;26(4):320–5	241	18.54	270	483	269	0
65	Yoshida Y, Yoshihara K, Nagaoka N, Hayakawa S, Torii Y, Ogawa T, Osaka A, Meerbeek BV. Self-assembled Nano-layering at the Adhesive interface. J Dent Res. 2012 Apr;91(4):376–81	235	21.36	263	489	270	0
66	Fukegawa D, Hayakawa S, Yoshida Y, Suzuki K, Osaka A, Van Meerbeek B. Chemical interaction of phosphoric acid ester with hydroxyapatite. J Dent Res. 2006 Oct;85(10):941–4	235	13.82	253	371	243	0
67	Uno S, Asmussen E. Marginal adaptation of a restorative resin polymerized at reduced rate. Scand J Dent Res. 1991 Oct;99(5):440–4	235	7.34	259	422	227	3
68	Sano H, Yoshiyama M, Ebisu S, Burrow MF, Takatsu T, Ciucchi B, Carvalho R, Pashley DH. Comparative SEM and TEM observations of nanoleakage within the hybrid layer. Oper Dent. 1995 Jul-Aug;20(4):160–7	234	8.36	246	388	183	0
69	Cadenaro M, Antoniolli F, Sauro S, Tay FR, Di Lenarda R, Prati C, Biasotto M, Contardo L, Breschi L. Degree of conversion and permeability of dental adhesives. Eur J Oral Sci. 2005 Dec;113(6):525–30	233	12.94	256	443	243	?
70	Shono Y, Ogawa T, Terashita M, Carvalho RM, Pashley EL, Pashley DH. Regional measurement of resin-dentin bonding as an array. J Dent Res. 1999 Feb;78(2):699–705	232	9.67	265	396	235	0
71	Cardoso MV, de Almeida Neves A, Mine A, Coutinho E, Van Landuyt K, De Munck J, Van Meerbeek B. Current aspects on bonding effectiveness and stability in adhesive dentistry. Aust Dent J. 2011 Jun;56 Suppl 1:31–44	231	19.25	248	580	236	7
72	Hikita K, Van Meerbeek B, De Munck J, Ikeda T, Van Landuyt K, Maida T, Lambrechts P, Peumans M. Bonding effectiveness of adhesive luting agents to enamel and dentin. Dent Mater. 2007 Jan;23(1):71–80	231	14.44	261	670	254	0
73	Ceballo L, Toledano M, Osorio R, Tay FR, Marshall GW. Bonding to Er-YAG-laser-treated dentin. J Dent Res. 2002 Feb;81(2):119–22	231	11	253	418	180	0
74	Tay FR, Gwinnett JA, Wei SH. Micromorphological spectrum from overdrying to overwetting acid-conditioned dentin in water-free acetone-based, single-bottle primer/adhesives. Dent Mater. 1996 Jul;12(4):236–44	229	8.48	258	460	231	?
75	Hanabusa M, Mine A, Kuboki T, Momoi Y, Van Ende A, Van Meerbeek B, De Munck J. Bonding effectiveness of a new 'multi-mode' adhesive to enamel and dentine. J Dent. 2012 Jun;40(6):475–84	225	20.45	254	541	258	0
76	Ausiello P, Apicella A, Davidson CL. Effect of adhesive layer properties on stress distribution in composite restorations–a 3D finite element analysis. Dent Mater. 2002 Jun;18(4):295–303	225	10.71	272	466	236	3
77	Van Meerbeek B, Yoshida Y, Lambrechts P, Vanherle G, Duke ES, Eick JD, Robinson SJ. A TEM study of two water-based adhesive systems bonded to dry and wet dentin. J Dent Res. 1998 Jan;77(1):50–9	225	9	234	406	194	0
78	Marshall SJ, Bayne SC, Baier R, Tomsia AP, Marshall GW. A review of adhesion science. Dent Mater. 2010 Feb;26(2):e11-6	224	17.23	252	496	234	3
79	Heintze SD, Rousson V. Clinical effectiveness of direct class II restorations—a meta-analysis. J Adhes Dent. 2012 Aug;14(5):407–31	222	20.18	239	457	242	15
80	Van Landuyt KL, Kanumilli P, De Munck J, Peumans M, Lambrechts P, Van Meerbeek B. Bond strength of a mild self-etch adhesive with and without prior acid-etching. J Dent. 2006 Jan;34(1):77–85. d	220	12.94	241	433	203	0
81	Armstrong S, Geraldeli S, Maia R, Raposo LH, Soares CJ, Yamagawa J. Adhesion to tooth structure: a critical review of "micro" bond strength test methods. Dent Mater. 2010 Feb;26(2):e50-62	219	16.85	245	569	258	?
82	Watts DC, Cash AJ. Determination of polymerization shrinkage kinetics in visible-light-cured materials: methods development. Dent Mater. 1991 Oct;7(4):281–7	219	6.84	241	408	222	12
83	Sanares AM, Itthagarun A, King NM, Tay FR, Pashley DH. Adverse surface interactions between one-bottle light-cured adhesives and chemical-cured composites. Dent Mater. 2001 Nov;17(6):542–56	217	10.33	246	446	222	3
84	Boschian Pest L, Cavalli G, Bertani P, Gagliani M. Adhesive post-endodontic restorations with fiber posts: push-out tests and SEM observations. Dent Mater. 2002 Dec;18(8):596–602	214	10.19	255	557	209	0
85	Choi KK, Condon JR, Ferracane JL. The effects of adhesive thickness on polymerization contraction stress of composite. J Dent Res. 2000 Mar;79(3):812–7	214	9.3	239	466	230	3
86	Martínez-Insua A, Da Silva Dominguez L, Rivera FG, Santana-Penín UA. Differences in bonding to acid-etched or Er:YAG-laser-treated enamel and dentin surfaces. J Prosthet Dent. 2000 Sep;84(3):280–8	213	9.26	232	428	241	0
87	Gwinnett AJ, Matsui A. A study of enamel adhesives. The physical relationship between enamel and adhesive. Arch Oral Biol. 1967 Dec;12(12):1615–20	213	3.8	227	507	230	1
88	Tay FR, Suh BI, Pashley DH, Prati C, Chuang SF, Li F. Factors contributing to the incompatibility between simplified-step adhesives and self-cured or dual-cured composites. Part II. Single-bottle, total-etch adhesive. J Adhes Dent. 2003 Summer;5(2):91–105	207	10.35	141	254	123	0
89	Yoshiyama M, Tay FR, Doi J, Nishitani Y, Yamada T, Itou K, Carvalho RM, Nakajima M, Pashley DH. Bonding of self-etch and total-etch adhesives to carious dentin. J Dent Res. 2002 Aug;81(8):556–60	206	9.81	224	435	205	0
90	Imazato S, Kinomoto Y, Tarumi H, Ebisu S, Tay FR. Antibacterial activity and bonding characteristics of an adhesive resin containing antibacterial monomer MDPB. Dent Mater. 2003 Jun;19(4):313–9	205	10.25	224	346	220	6
91	Van Noort R, Cardew GE, Howard IC, Noroozi S. The effect of local interfacial geometry on the measurement of the tensile bond strength to dentin. J Dent Res. 1991 May;70(5):889–93	205	6.41	211	355	175	?
92	Imazato S. Bio-active restorative materials with antibacterial effects: new dimension of innovation in restorative dentistry. Dent Mater J. 2009 Jan;28(1):11–9	204	14.57	218	311	232	0
93	Carvalho RM, Chersoni S, Frankenberger R, Pashley DH, Prati C, Tay FR. A challenge to the conventional wisdom that simultaneous etching and resin infiltration always occurs in self-etch adhesives. Biomaterials. 2005 Mar;26(9):1035–42	204	11.33	227	431	214	0
94	Oliveira SS, Pugach MK, Hilton JF, Watanabe LG, Marshall SJ, Marshall GW Jr. The influence of the dentin smear layer on adhesion: a self-etching primer vs. a total-etch system. Dent Mater. 2003 Dec;19(8):758–67. d	204	10.2	236	432	205	0
95	Chen C, Niu LN, Xie H, Zhang ZY, Zhou LQ, Jiao K, Chen JH, Pashley DH, Tay FR. Bonding of universal adhesives to dentine–Old wine in new bottles? J Dent. 2015 May;43(5):525–36	202	25.25	229	406	206	3
96	Fusayama T, Nakamura M, Kurosaki N, Iwaku M. Non-pressure adhesion of a new adhesive restorative resin. J Dent Res. 1979 Apr;58(4):1364–70	201	4.57	241	691	249	0
97	Peumans M, De Munck J, Van Landuyt KL, Poitevin A, Lambrechts P, Van Meerbeek B. Eight-year clinical evaluation of a 2-step self-etch adhesive with and without selective enamel etching. Dent Mater. 2010 Dec;26(12):1176–84	199	15.31	216	425	211	0
98	Yiu CK, Pashley EL, Hiraishi N, King NM, Goracci C, Ferrari M, Carvalho RM, Pashley DH, Tay FR. Solvent and water retention in dental adhesive blends after evaporation. Biomaterials. 2005 Dec;26(34):6863–72	199	11.06	219	341	204	1
99	Yoshikawa T, Sano H, Burrow MF, Tagami J, Pashley DH. Effects of dentin depth and cavity configuration on bond strength. J Dent Res. 1999 Apr;78(4):898–905	199	8.29	221	408	204	?
100	Breschi L, Maravic T, Cunha SR, Comba A, Cadenaro M, Tjäderhane L, Pashley DH, Tay FR, Mazzoni A. Dentin bonding systems: From dentin collagen structure to bond preservation and clinical applications. Dent Mater. 2018 Jan;34(1):78–96	198	39.6	222	343	238	0
101	Spencer P, Wang Y, Walker MP, Wieliczka DM, Swafford JR. Interfacial chemistry of the dentin/adhesive bond. J Dent Res. 2000 Jul;79(7):1458–63	198	8.61	208	276	184	0

More recent articles were listed with priority for articles with the same numbers of citations. The list of journal names was arranged in order of their numbers of top-cited articles, and the Journal Impact Factor (JIF) 2021 from the Journal of Citation Reports (https://jcr.clarivate.com) was used to rate journals with the same numbers of articles (Table S2). The institute of origin was based on the address of the first author's affiliation. If the first author worked at more than one institution that belonged to more than one country, each institution and country were counted. The type of study was classified as clinical, basic, review, systematic review, meta-analysis, or lecture based on the article type. To determine the area of study, the full text of each article was carefully examined by identifying concepts based on MeSH terms from PubMed.

The Visualization of Similarities (VOS) Viewer software program (version 1.6.15; Centre for Science and Technology Studies, Leiden University) was used to analyze the co-authorship network and journals. SPSS version 21 (IBM Corporation, USA) was used for the statistical analysis of the frequencies of the descriptive measures.

## Results

The top 100 most-cited articles are listed in Table 1 according to the number of citations. The most cited article, published in 2003 by Van Meerbeek et al. in Operative Dentistry, had 1288 citations and was a lecture on adhesion to enamel and dentin (Table 1). The least-cited article had 198 citations. The top 100 most cited articles had a total of 34,526 citations, and the mean number of citations per article was 342.

### Journals and years of publication

**Fig. 1:**
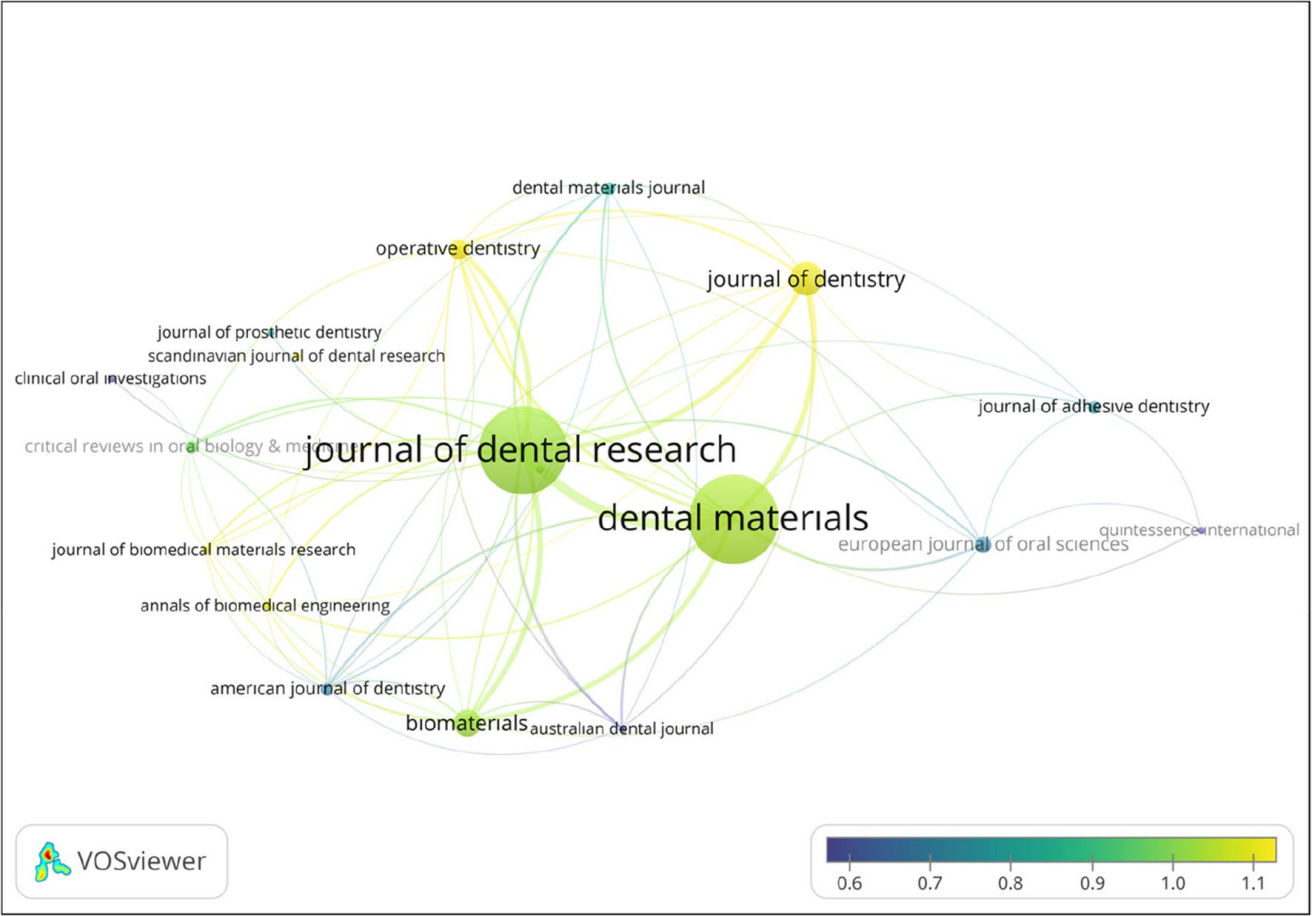
Journal citation map of the 101 most cited articles; bubbles indicate the number of publications and colour indicates the average normalised citation. (Using VOSviewer interface, in the 'Overlay Visualisation' section, "LinLog/modularity" was selected as the analysis method, the number of articles contributed by the journals as 'Weights' and the average normalised citation count as ‘Scores’)

**Fig. 2:**
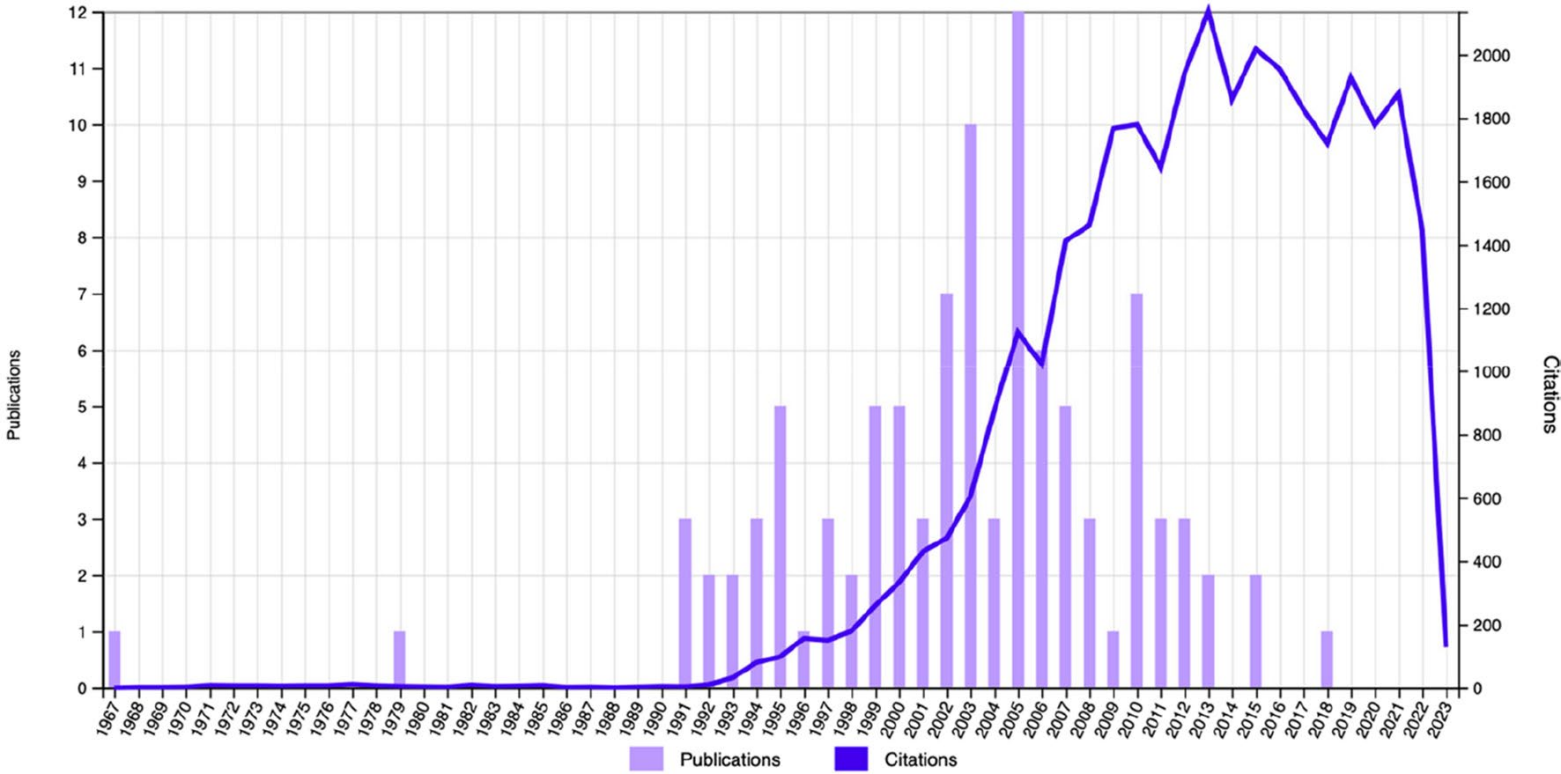
The number of articles by years and the total number of citations of the top 100 articles by years

The top 100 cited articles were published in 18 journals, all in the English language. Nine of the 18 journals had each published only one of the 100 most cited articles, while three other journals had each published two articles. The other 6 journals that published at least 3 of the most cited articles are shown in Fig. 1. The impact factors of the six journals were between 2.16 and 15.304. The journal with the highest number of top-cited articles (*n* = 33) was the Journal of Dental Research, followed by Dental Materials (*n* = 32) and the Journal of Dentistry (*n* = 8). The top 100 most cited articles were published between 1967 and 2018 (Fig. 2). Sixty-two of these articles were published between 2000 and 2010. The year 2005 had the highest number of top-cited articles (*n* = 12), followed by 2003 (*n* = 10), 2002, and 2010 (*n* = 7). The oldest article, written by Gwinnett et al., was published in the Archives of Oral Biology in 1967. The newest article was written by Breschi et al. and published in Dental Materials in 2018.

### Authors, countries, and institutions of origin

**Fig. 3:**
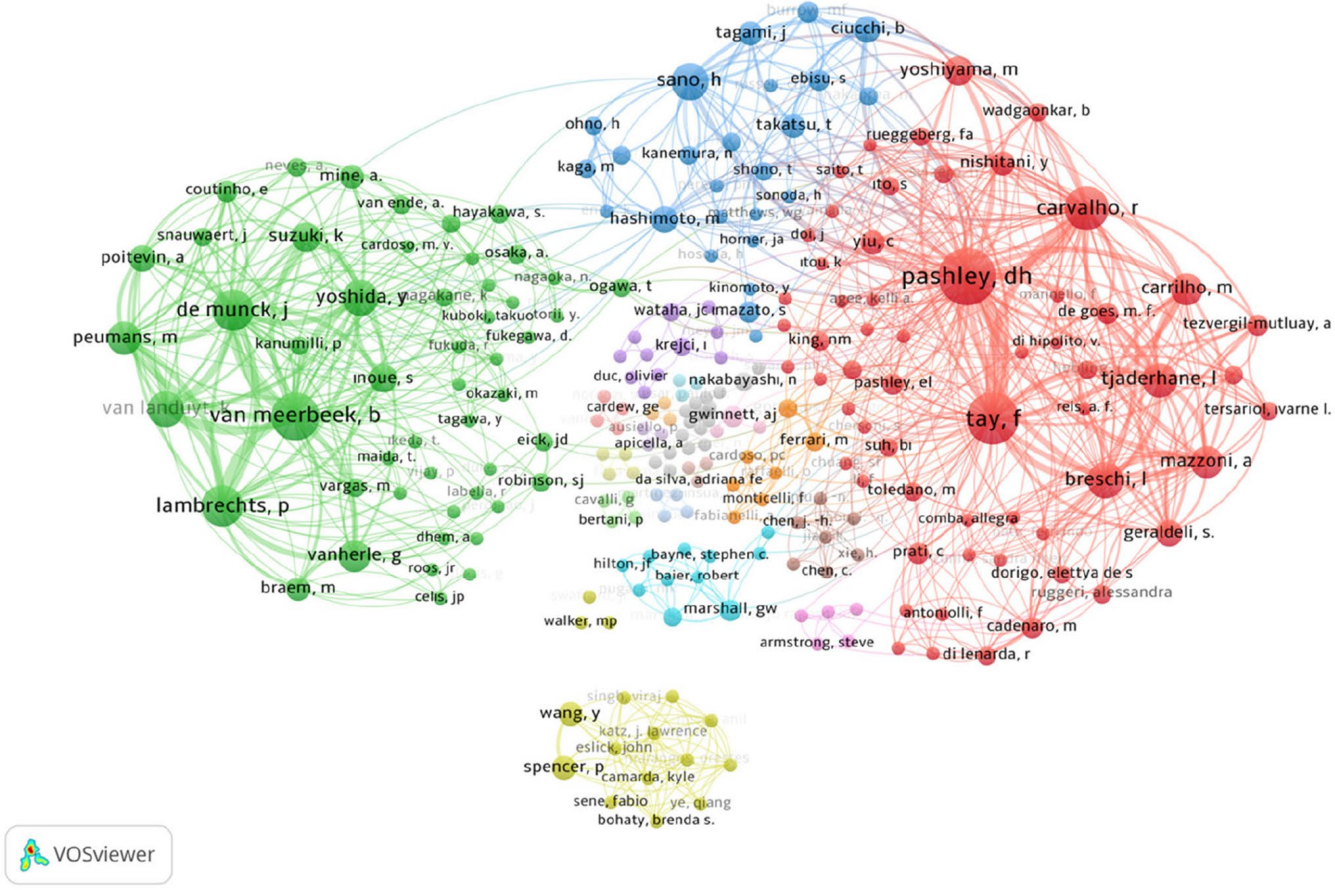
A co-authorship map shows all the contributor authors of the 101 top-cited articles. From VOSviewer interface; in analysis option “LinLog/modularity” selected as normalization method and in the ‘Weights’ drop-down list from Visualization section, ‘Documents’ option was selected to determine the label sizes of the authors depending on the number of articles to which they contributed

In total, 244 unique authors contributed to the 100 most cited articles. Five articles were attributed to a single author; 13 articles to two authors; and 83 articles to three or more authors. The top 100 list consisted of 65 different first authors. The most cited articles were published by Van Meerbeek B. (9 articles; 4650 citations), followed by those by Pashley D.H. (6 articles; 2769 citations) and Tay F.R. (6 articles; 2184 citations) (Table 2). Regarding the total author network, Pashley D.H. was leading with 37 articles and 12517 citations, followed by Tay F.R. (30 articles; 9607 citations), Van Meerbeek B. (24 articles; 11,088 citations), Carvalho R.M. (19 articles; 6804 citations), De Munck J. (16 articles; 8444 citations), and Lambrechts P. (16 articles; 7811 citations; Fig. 3).

**Table 2:** First authors with three or more top-cited articles

Authors	No. of articles	No. of citations*	Citations per article*	H index*
Van Meerbeek, B	9	4620	513,33	72
Pashley, DH	6	2769	461,5	77
Tay, FR	6	2184	364	91
Sano, H	5	1952	390,4	55
Van Landuyt, KL	3	1417	472,33	51
Breschi, L	3	1261	420,33	58
Spencer, P	3	830	276,67	45
Imazato, S	3	735	245	47

^*^for WOS

The first author's address was used to ascertain the country of origin. Accordingly, the top 100 articles originated from 16 countries (Table 3), of which Japan had the highest number of articles (25 articles; 7847 citations), followed by Belgium (20 articles; 9572 citations), the United States (18 articles; 5805 citations), Italy (9 articles; 2784 citations), and Brazil (6 articles; 1883 citations).

**Table 3:** Countries with two or more top-cited articles

Countries	No. of articles	No. of citations*	Citation per year*
Japan	25	7847	313.88
Belgium	20	9572	478.6
USA	18	5805	322.5
Italy	9	2784	309.33
Brazil	6	1883	313.83
Hong Kong	5	1629	325.8
China	3	966	322
Switzerland	3	905	301,67
Liechtenstein	2	679	339.5
Finland	2	542	271
Spain	2	444	222
UK	2	424	212

^*^for WoS

On the basis of the first authors' addresses, 38 institutions contributed to the top 100 most cited publications, of which 10 had at least 3 publications (Table 4). Among the 10 institutions, the most contributions were made by the Catholic University of Leuven (20 articles; 9572 citations), followed by Tokyo Medical and Dental University (10 articles; 3118 citations), the University of Hong Kong, and Prince Philip Dental Hospital (7 articles; 2393 citations).

**Table 4:** Institutions with three or more top-cited articles

Institutions	No. of articles	No. of citations*	Citation per year*
Catholic University of Leuven	20	9572	478.6
Tokyo Medical and Dental University	10	3118	311.8
University of Hong Kong, Prince Philip Dental Hospital	7	2393	341.86
Hokkaido University	6	2042	340.33
Medical College of Georgia	6	2769	461.5
Okayama University	5	1720	344
University of Missouri-Kansas City	4	1081	270.25
University of Trieste	3	1296	432
Osaka University	3	735	245
University of Geneva	3	905	301.67

^*^for WoS

### Type and field of study

With 69 articles, basic science research had the highest number of articles among the top 100 most cited articles. Twenty-five articles were reviews, 3 articles were systematic reviews, 1 article was a meta-analysis, 1 article was a systematic review and meta-analysis, 1 article was a lecture, and 2 articles reported clinical trials (Table 5). One of the two clinical trials included both in vivo and in vitro studies. The major topic of interest in the top 69 most cited basic science articles was the ultramorphological structures of dentin and adhesive interfaces (39 articles), followed by bond strength to dentin (34 articles) and hybrid layers (25 articles). The major topic of interest in the top 25 most cited review articles was the hybrid layer (11 articles), followed by the ultramorphological structures of dentin and adhesive interfaces (8 articles) and bonding to dentin (7 articles). Of the two clinical studies, one was related to the clinical performances of total-etch adhesive systems, and the other was on the clinical performance of multimode adhesive systems (Table 5).

**Table 5:** Numbers of the top-cited articles categorized on basis of type and specific field

Field of study	Type of study						
	Clinical	Basic	Review	Systematic review and Meta analysis	Meta analysis	Systematic review	Lecturer
	**2**	**69**	**25**	**1**	**1**	**3**	**1**
Acid etching	1	10	3	1			1
Etch and rinse adhesives		13	5	1	1	2	1
Self-etch adhesives		12	5		1	3	1
Multi-mode adhesives	1	1					
Universal adhesives		1					
Glass ionomer adhesives			2				1
Dentin adhesive / Micro-Bond Strength Testing			4				1
Dentin adhesive / Micro-shear testing		1	4				
Dentin adhesive / Macro-tensile/push-out		1	2				
Bonding to Enamel		1	2				1
Bonding to Dentin		1	7				1
Dentin adhesive / Durability		2	6				
Dentin adhesive / Aging		4	3	1			1
Dentin adhesive / Degradation		9	4				
Dentin adhesive / MMP inhibitors		4	3				
Dentin adhesive / Collagen cross-linking			2				
Dentin adhesive / Chemistry		1				2	
Dentin adhesive / Classification			3				1
Dentin adhesive / Hybrid layer		25	11				1
Dentin adhesive / Nanoleakage		6	3				1
Dentin adhesive / Sealing effectiveness		3	2				1
Dentin adhesive / Microleakage		3	2				
Dentin adhesive / Bond strength to enamel		8	1	1			1
Dentin adhesive / Bond strength to dentin		34	5	1			1
Dentin adhesive / Push out strength		1					
Dentin adhesive / Bond strength to composite		1	1				
Dentin adhesive / Diametral compressive strength		1					
Dentin adhesive / Fatigue			1				
Dentin adhesive / Fatigue strength			2				1
Dentin adhesive / Clinical performance	2		4		1	2	
Dentin adhesive / Nano-layer		1	1				
Dentin adhesive / Smear layer		4	6				
Dentin adhesive / Ultramorphological structure of dentin adhesive interface		39	8				
Dentin adhesive / Microanalysis		2	1				
Dentin adhesive / Functional monomers		3					
Dentin adhesive / Collagenolytic activity of dentin (host-derived enzymes)		4	5				
Dentin adhesive / Wet bonding		6	4				
Dentin adhesive / Dry bonding		4	1				
Dentin adhesive / Phase separation		2	1				
Dentin adhesive / Solvent		1	1				
Dentin adhesive / Relationship between laboratory and clinical bonding effectiveness			1				
Dentin adhesive / Resin–dentin interdiffusion zone		3					
Dentin adhesive / Monomer convertion		3					
Dentin adhesive / Water sorption & solubility		3	1				
Dentin adhesive / Modulus of elasticity		4					
Dentin adhesive / Hardness		1					
Dentin adhesive / Microhardness		1					
Dentin adhesive / Dentin permeability		1	2				
Dentin adhesive / Polymerization shrinkage		4					
Dentin adhesive / Antibacterial properties		1	2				
Dentin adhesive / Biocompatibility		1	2				
Dentin adhesive / Hypersensitivity			2				
Dentin adhesive / Remineralization			2				
Dentin adhesive/pH		1					

### Altmetric assessment

Among the top 100 most-cited articles, 43 had AASs. Forty-nine articles had interactions that were not mentioned, and nine had interactions that were not included in the calculation of the AAS. The AASs of the 43 articles were as follows: 1–5 in 27 articles, 6–10 in 12, and 10 or higher in 4. The article with the highest AAS (24), a review on dentin adhesive/aging written by Breschi et al., was published in Dental Materials in 2008. This is followed by a meta-analysis on clinical performance written by Heintze et al. and published in the Journal of Adhesive Dentistry in 2012 (AAS = 15).

## Discussion

In our study, the citation count of the top 100 articles was between 1288 and 198 on WoS, between 1464 and 208 on Scopus, between 3118 and 276 on Google Scholar, and between 1100 and 184 on Dimensions. The total number of citations was highest on Google Scholar, followed by Scopus, WoS, and Dimensions. However, the number of citations for the same article differed between the databases. In addition to scientific articles, Google Scholar includes citations from books, theses, and other works, so the results from the database should be interpreted with caution. Currently, Scopus only counts citations from 1996 onwards, which is a major shortcoming for identifying the most cited journal articles, but expansion of the citation count to before 1996 has been planned for the near future [29]. On the other hand, the database indexes more international and open-access journals than the WoS [5]. In accordance with another study, the Dimensions database was assessed using a free application that does not provide entry to the website's functionalities, including grants, patents, clinical trials data, and analytical tools [7]. By contrast, citations were collected using the complete versions of WoS and Scopus [46]. Moreover, while the number of citations on WoS, Scopus, and Dimensions showed no correlation with the AASs, the number of citations on Dimensions strongly correlated with those on WoS and Scopus. Both Dimensions and Altmetric can provide a more comprehensive assessment of research effects [46]. In parallel with the main logic of our study, the “all databases” section of the ISI Web of Knowledge database was selected as the main database in other studies because it can count citations in scientific articles over a wide period from 1945 to the present [12, 22, 29]. In our study, the number of citations was lower than those in studies conducted in different dentistry areas such as endodontics (between 2115 and 246 citations) [17] and implant dentistry (between 2229 and 199 citations) [19], but higher than those in other studies on dentistry areas such as pediatric dentistry (between 182 and 42 citations) [22], oral medicine and radiology (between 624 and 86 citations) [26], and orthodontics (between 545 and 89 citations) [28]. In fact, the citation rates differed for each specialization depending on the number of researchers working in a specific field [15]. In addition, the wide variety of subdisciplines in specific fields may be another influencing factor in the citation rate.

Of the most cited articles in our study, 89.1% (90 articles) were published before 2010. Our findings are consistent with those of other studies [15, 17, 19, 28, 29]. Contrary to our findings, the most cited articles in some studies were published in the past decade [11, 12, 22]. The oldest articles have more time to be cited than recent articles, regardless of their scientific significance, hence the risk of exemption from the recent influential articles [25]. As supported by our findings and previous studies, it can be considered that an article needs a publication period of at least 6 to 15 years to receive sufficient citations and become a citation classic [19]. This may explain why none of the 100 most cited articles in our study were published in the last 5 years. According to Kuhn's philosophy, the scientific community has a tendency to stick to a paradigm[47]. In this context, this means that citations have a “snowball effect” because other authors are more inclined to cite articles on the basis of their numbers of earlier citations and not their content or quality [48]. On the other hand, a publication with more than 400 citations should be considered a classic, but in some areas where researchers have fewer, 100 citations may merit a study [15, 49]. The first 13 most cited articles in our study were cited more than 400 times, whereas the 100th article was cited 198 times. Therefore, in our study, the attribution rate was influenced not just by the snowball effect but also by the article's content or quality. Moreover, when the AASs were analyzed, the rate of mentioning articles published after 2010 on social media was 23.3% (8 articles), which is higher than the citation rates. One study found a high correlation between the citation count in Dimensions and those in WOS and Scopus but found no correlation between the citation counts in WOS, Scopus, Dimensions, and Altmetric [46]. Another study reported a weak but positive correlation between the AAS and the number of citation [50]. In Altmetric, behavior is completely different from the classic citation system, allowing recently published works to achieve more recognition and visibility quickly. Thus, Altmetric can highlight newly published research articles with higher prevalence rather than top-cited articles, which are usually at least 1 or 2 decades old [46].

In our study, 70% of the first 100 most cited articles (71 articles) were published in journals with an impact factor greater than 5 and a high impact factor for the field of dentistry. Except for one, the other 29 most cited articles were published in journals with impact factors higher than 2, of which 15 were in journals with impact factors greater than 3, which indicates a relatively high impact. This result was consistent with those of other studies [4, 24, 35]. It is well known that researchers choose high-impact journals for their article submissions and that journals with high impact factors attract high-quality articles [26]. However, no correlation was found between the journal impact factor and the number of articles that received the most citations [17, 26]. On the contrary, the number of citations and the relevant impact factor have been found to be closely correlated in a limited number of journals, especially in areas with high citation intensity [4, 24]. This can be attributable to the fact that articles with high citation rates tend to be published in journals with high impact factors [35]. In addition, more than a third of the articles have been published in specialty journals, including the subjects of our study, and this result may justify why fewer journals have attracted more attention [26]. Therefore, this conforms to Bradford's law, which explains why only a few journals in a subject area are most frequently cited and consequently most likely to be of interest to researchers in the discipline [22, 50, 51]. In line with our findings, similar results have been observed in other studies [17, 25, 26].

This study shows that 25 of the 100 most cited articles originated in Japan. The introduction of resin bonding to etched dentin by Fusuyama et al. [41], along with extensive research conducted in the following decade, and later, the definition of hybrid layer by Nakbayashi [52], had a significant influence on most of the highly cited articles, all of which had Japanese origins. In our study, 20 of the 100 most cited articles were affiliated with the Catholic University of Leuven in Belgium and were published between 1992 and 2012. This was followed by 10 articles from Tokyo Medical and Dental University, spanning the years 1979 to 1999, and 7 articles from the University of Hong Kong, Prince Philip Dental Hospital, covering the period between 1996 and 2005. These universities are particularly focused on the subspecialty of dental adhesion. Remarkably, although nearly one-fifth of the 100 most cited articles were produced by institutions in Japan, the most cited articles were from Belgium (Catholic University of Leuven), particularly considering that Japanese articles were among the earliest and most pioneering contributions to the field. Despite Belgium's modest population, researchers from this country have been comparatively prolific in operative dentistry-related publications during the study period [29, 53], aligning with our finding that researchers affiliated with this center had two or more highly referenced articles (Fig. 3). Also, the reasons for the high citation rates of Belgian articles could be attributed to factors such as international collaboration, research infrastructure, and visibility within the global scientific community. In addition, in line with the results of other studies [15, 17], approximately one-third of the most cited articles (28 articles) in our study were produced by independent institutions. It's essential to consider the extent of international collaboration in dentin adhesive research. Articles resulting from collaborative efforts between researchers from various countries might have received more citations due to their diverse perspectives and broad relevance.

In our study, most of the top-cited articles were in the field of basic research (69 articles), followed by reviews (25 articles) and systematic review and/or meta-analysis (5 articles). Only two of the top cited articles reported clinical experiences. Consistent with our findings, other studies have reported that most of the top-cited articles were in the field of basic science [15, 17, 39]. On the other hand, other studies found that most top-cited articles reported clinical experiences [4, 19, 25, 28]. However, one study found that the most top-cited articles were reviews [13]. These differences may be due to differences in subspecialties in the field of dentistry. Most of the top-cited articles in our study were in the field of basic science. In the early stages of dentin adhesive development, the papers that formed the foundation of the field generally focused on basic research, investigating the principles of adhesion, the composition of adhesives, and their interactions with dentin. Some of the pioneering articles from this period, while groundbreaking, may have been more cited because of their age. Basic research in dentin adhesives, a subspecialty of operative dentistry, is crucial to investigating the efficacy of new materials or modified techniques [15]. In vitro studies play an important role in enhancing methods and providing early data on which later research with greater evidence can be based [11]. In our study, most topics in basic science were on the ultramorphological structures of dentin and adhesive interfaces (39 articles), followed by bond strength to dentin (34 articles) and hybrid layers (25 articles). The integration of knowledge from new basic science research into the subspecialty practices of operative dentistry provides the opportunity to address major clinical issues [15]. However, the fact that our study detected very few systematic reviews, meta-analyses, and RCTSs among the most cited papers suggests that more such studies on dentin adhesives are needed.

As with other citation analyses, this study has some limitations. By including many databases, the differences in the number of citations between databases were tried to be eliminated. The current study excluded several articles, as indicated by the title, due to its focus on including only the top 100 most-cited articles. In addition, articles written in languages other than English and books or conference proceedings as document type were not included in the study.

## Conclusion

Most top-cited articles (89.1%) were published before 2010. In our study, the most frequently cited articles were concentrated in a few journals. As first author, Van Meerbeek B. has the highest number of articles with nine articles and a total number of 4650 citations. The highest top-cited 100 articles originated from Japan. The most top-cited articles originated from the Catholic University of Leuven in Belgium. Basic science research had the highest number of articles, followed by reviews. The primary foci of basic research were the ultramorphological structures of dentin and adhesive interfaces. The major topic of the reviews was hybrid layers. Only 2 RCTs and a few systematic reviews and meta-analyses were published. Thus, in the future, studies with high levels of evidence, such as systematic reviews, meta-analyses, and RCTs, are required.

### Supplementary Information

Below is the link to the electronic supplementary material.

Supplementary file1 (DOCX 17 KB)

Supplementary file2 (DOCX 18 KB)
