# Supplementary material for: A bibliometric and Altmetric analysis of the 100 top most cited articles on dentin adhesives
Source: Clin Oral Investig. 2024 Jan 13;28(1):92. doi: 10.1007/s00784-024-05498-5 (PMC10787682; doi:10.1007/s00784-024-05498-5)
Supplement: Supplementary file 1 — Supplementary file1 (DOCX 17 KB) [file 784_2024_5498_MOESM1_ESM.docx]

**Table S1** Search strategy used in ISI Web of Knowledge (12.02.2023)

| Search terms |
| --- |
| TS=(Adhesive* OR Dentin adhesive* OR Total-etch adhesive* OR Total-etch adhesive system* OR Total-etch OR Total-etching OR Etch and rinse adhesive* OR Etch-and-rinse adhesive* OR Etch-&-rinse adhesive* OR Three-step etch and rinse adhesive* OR Two-step etch and rinse adhesive* OR 3-step etch and rinse adhesive* OR 2-step etch and rinse adhesive* OR two-bottle etch-and-rinse adhesive* OR two-bottle total-etch adhesive* OR two-bottle total-etch adhesive system* OR one-bottle etch –and-rinse adhesive* OR one-bottle etch and rinse adhesive* OR etch-and-rinse single bottle adhesive* OR One bottle total etch adhesive OR One bottle total etch adhesive system* OR Self-etch adhesive* OR Self-etch adhesive system* OR Self-etch OR self-etching primers OR self-etching adhesive* OR Two-step self-etch adhesive* OR 2-step self-etch adhesive* OR two-bottle self-etch adhesive* OR All-in-one adhesive* OR One-bottle adhesive* OR one-bottle self-etching adhesive* OR 1-step self-etch adhesive* OR one-step self-etch adhesive* OR one-bottle simplified adhesive* OR Universal adhesive* OR Universal dental adhesive* OR Universal simplified adhesive system* OR Universal dental adhesive system* OR Multipurpose adhesive* OR Multi-purpose adhesive* OR Multimode adhesive* OR One Bottle Multi-mode Adhesive OR Glass-ionomer adhesive* OR Amalgam Adhesive* OR Ceramic adhesive* OR Resin-modified glass-ionomer adhesive* OR 1st generation dentin adhesive* OR 2nd generation dentin adhesive* OR 3nd generation dentin adhesive* OR 4th generation dentin adhesive* OR 5th generation dentin adhesive* OR 6th generation dentin adhesive* OR 7th generation dentin adhesive* OR 8th generation dentin adhesive* OR First generation dentin adhesive* OR Second generation dentin adhesive* OR Third generation dentin adhesive* OR Fourth generation dentin adhesive* OR Sixth generation dentin adhesive* OR Seventh generation dentin adhesive* OR Eighth generation dentin adhesive* OR Dentin Bonding Agent* OR Dentin-bonding agent* OR Total-etch dentin bonding agent* OR Total-etch dentin bonding agent* OR Etch and rinse dentin bonding agent* OR Etch-and-rinse dentin bonding agent* OR Etch-&-rinse dentin bonding agent* OR Three-step etch and rinse dentin bonding agent* OR Two-step etch and rinse dentin bonding agent* OR 3-step etch and rinse dentin bonding agent* OR 2-step etch and rinse dentin bonding agent* OR two-bottle etch-and-rinse dentin bonding agent* OR two-bottle total-etch dentin bonding agent* OR one-bottle etch –and-rinse dentin bonding agent* OR one-bottle etch and rinse dentin bonding agent* OR etch-and-rinse single bottle dentin bonding agent* OR One bottle total etch dentin bonding agent* OR Self-etch dentin bonding agent* OR Self-etch OR self-etching primers OR self-etching dentin bonding agent* OR Two-step self-etch dentin bonding agent* OR 2-step self-etch dentin bonding agent* OR two-bottle self-etch dentin bonding agent* OR All-in-one dentin bonding agent* OR One-bottle dentin bonding agent* OR one-bottle self-etching dentin bonding agent* OR 1-step self-etch dentin bonding agent* OR one-step self-etch dentin bonding agent* OR one-bottle simplified dentin bonding agent* OR Universal dentin bonding agent* OR Universal simplified dentin bonding agent* OR Multipurpose dentin bonding agent* OR Multi-purpose dentin bonding agent* OR Multimode dentin bonding agent* OR One Bottle Multi-mode dentin bonding agent* OR Glass-ionomer dentin bonding agent* OR Amalgam dentin bonding agent* OR Ceramic bonding agent* OR Resin-modified glass-ionomer dentin bonding agent* OR 1st generation dentin bonding agent* OR 2nd generation dentin bonding agent* OR 3nd generation dentin bonding agent* OR 4th generation dentin bonding agent* OR 5th generation dentin bonding agent* OR 6th generation dentin bonding agent* OR 7th generation dentin bonding agent* OR 8th generation dentin bonding agent* OR First generation dentin bonding agent* OR Second generation dentin bonding agent* OR Third generation dentin bonding agent* OR Fourth generation dentin bonding agent* OR Sixth generation dentin bonding agent* OR Seventh generation dentin bonding agent* OR Eighth generation dentin bonding agent*) |
