# Supplementary material for: A bibliometric and Altmetric analysis of the 100 top most cited articles on dentin adhesives
Source: Clin Oral Investig. 2024 Jan 13;28(1):92. doi: 10.1007/s00784-024-05498-5 (PMC10787682; doi:10.1007/s00784-024-05498-5)
Supplement: Supplementary file 2 — Supplementary file2 (DOCX 18 KB) [file 784_2024_5498_MOESM2_ESM.docx]

**Table S2** Journals containing top cited articles

| Journal Title |  | IF (2021) | No. Of Articles |
| --- | --- | --- | --- |
| Journal of Dental Research  (Critical Reviews in Oral Biology & Medicine) |  | 8.924 | 33 |
| Dental Materials |  | 5.687 | 32 |
| Journal of Dentistry |  | 4.991 | 8 |
| Biomaterials |  | 15.304 | 6 |
| Operative Dentistry |  | 2.937 | 4 |
| European Journal of Oral Sciences |  | 2.16 | 3 |
| Journal of Adhesive Dentistry |  | 4.309 | 2 |
| Dental Materials Journal |  | 2.418 | 2 |
| American Journal of Dentistry |  | 1.748 | 2 |
| Annals of Biomedical Engineering |  | 4.219 | 1 |
| Journal of Prosthetic Dentistry |  | 4.148 | 1 |
| Journal of The American Dental Association |  | 3.681 | 1 |
| Journal of Biomedical Materials Research |  | 3.652 | 1 |
| Clinical Oral Investigations |  | 3.607 | 1 |
| Archives of Oral Biology |  | 2.64 | 1 |
| Australian Dental Journal |  | 2.259 | 1 |
| Quintessence International |  | 2.175 | 1 |
| Scandinavian Journal of Dental Research |  | - | 1 |
